# Prevalence, risk factors, and prognostic implications of intraoperative bleeding during CF-LVAD implant

**DOI:** 10.1016/j.jhlto.2024.100195

**Published:** 2024-12-13

**Authors:** Ibrahim Mortada, Christos Kourek, Rupesh Kshetri, Arun Singhal, Anthony Panos, Alexandros Briasoulis, Mohammed Mhanna, Shareef Mansour, Kristine Yumul, Paulino Alvarez, Ernesto Ruiz Duque

**Affiliations:** a Division of Cardiology, Department of Medicine, University of Iowa, Iowa City, Iowa; b Medical School of Athens, National and Kapodistrian University of Athens, Athens, Greece; c Department of Cardiovascular Surgery, University of Iowa, Iowa City, Iowa; d Department of Cardiovascular Medicine, Cleveland Clinic, Cleveland, Ohio; e Cleveland Clinic Lerner College of Medicine of Case Western Reserve University, Cleveland, Ohio

**Keywords:** LVAD, heart failure, bleeding, mechanical circulatory support, survival

## Abstract

**Background:**

The use of continuous flow left ventricular assist device (CF-LVAD) has revolutionized the management of advanced heart failure. One of the major complications associated with its use is the risk of bleeding, especially in the early postoperative period. Early events of postoperative bleeding have been associated with higher morbidity and mortality rates. Our study aims at identifying potential predictors of intraoperative bleeding, defined as 4 or more units of packed red blood cells transfused during surgery. A single-center retrospective cohort study of adult patients older than 18 years old who underwent CF-LVAD implantation between 2009 and 2024.

**Methods:**

Data were collected for the duration of implant hospitalization, including perioperative invasive hemodynamics, echocardiography, operative details, mechanical circulatory support, antiplatelets, inotropes, bleeding events, and blood product use, in addition to patient history and baseline characteristics.

**Results:**

A total of 208 patients were included in the analysis. Intraoperative bleeding occurred in 43 (20.67%) patients while 165 (79.33%) patients did not experience bleeding. Multilogistic regression analysis showed that artery bypass grafting pre-LVAD (odds ratio [OR] 2.98, confidence interval [CI] 1.2-7.42, *p* = 0.01) and temporary mechanical assist device pre-LVAD (OR 3.67, 95%CI 1.72-7.85, *p* < 0.001) were independent predictors of intraoperative bleeding during hospitalization. Intraoperative bleeding is also correlated with worse clinical outcomes, higher 90-day mortality (hazard ratio [HR] 10.4, *p* < 0.01, CI 95% 3.28-33.38) 206 subjects with 14 failures.

**Conclusion:**

History of coronary artery bypass grafting and mechanical circulatory support before the implantation of LVAD are independent predictors of intraoperative bleeding during hospitalization in these patients. Intraoperative bleeding is associated with higher frequency of right ventricle failure post-LVAD and higher 90-day mortality.

## Background

Continuous flow left ventricular assist device (CF-LVAD) has become the standard of care for selected patients with advanced heart failure with a 2-year survival rate of 76.9%.^1^ Despite a significant reduction of hemocompatibility–related adverse events, bleeding complications remain a significant source of morbidity and mortality. According to Interagency Registry for Mechanically Assisted Circulatory Support (INTERMACS) data, the most frequent locations of the first bleeding after LVAD implantation are mediastinal (45%), followed by thoracic pleural space (12%), gastrointestinal tract (18%), and chest wall (8%).^2^, ^3^ Among those bleeding in the early postoperative period necessitating surgery occurred in 10% to 18% of patients in randomized clinical trials.^2^, ^3^ The MOMENTUM 3 and ENDURANCE trials revealed elevated rates of early bleeding in each of the HeartMate II (HM II), HeartWare Ventricular Assist Device (HVAD), and HeartMate 3 (HM3) LVAD, necessitating surgery in 10% to 18% of patients.^4^, ^5^ Early events of postoperative bleeding have been associated with a high patient morbidity and mortality rates,^6^, ^7^ higher blood transfusion requirements,^8^ as well as higher cost of health care.^9^ An observational analysis from the MOMENTUM 3 trial showed that usual dose aspirin (325 mg daily) achieved a similar degree of bleeding and thrombotic events in HM3 LVAD when compared with low-dose aspirin (81 mg daily).^10^ The ARIES-HM3 randomized clinical trial concluded that in patients with advanced heart failure treated with a fully magnetically levitated LVAD, avoidance of aspirin as part of an antithrombotic regimen, which includes vitamin K antagonists, is not inferior to a regimen containing aspirin, does not increase thromboembolism risk, and is associated with a reduction in bleeding events.^11^ Use of preoperative antiplatelets is considered as a risk factor for increased perioperative bleeding and blood transfusion.^12^ Data regarding the association of preoperative antiplatelets and intraoperative outcomes in patients receiving LVAD still remains limited. There has been limited literature exploring the risk factors of intraoperative bleeding (IOB), including preoperative antiplatelets. Our study aims at evaluating the prevalence, risk factors, and prognostic implications of IOB during LVAD implantation.

## Methods

### Study design

This is a single-center retrospective cohort study of patients implanted with CF-LVAD from March 31, 2009 to May 1, 2024, at the University of Iowa Health Care. The study was approved by the local institutional review board. Patients were identified retrospectively through use of Slicer Dicer tool in Epic electronic medical record.

### Study population

**Figure 1:**
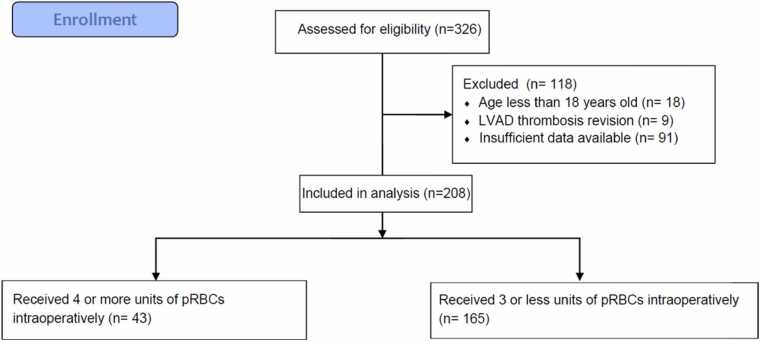
Flowchart of patients’ enrollment and exclusion. LVAD, left ventricular assist device; pRBCs, packed red blood cells.

Patients were included if they were age of 18 years or older and had received a CF-LVAD. Following review, a total of 326 patients were identified, and 208 patients did meet the inclusion criteria. Patients were excluded if they have had previous LVAD implant, or no data was available (Figure 1). Patient data extracted from the medical record included baseline cardiac risk factors, cardiac history, baseline cardiac testing, use of antiplatelets 5 days before surgery, chest open, chest exploration, and need for transfusions. We define IOB as the requirement of 4 or more units of packed red blood cells during surgery.^3^

### Surgical approach

All operations were performed in a standard manner with median sternotomy and cardiopulmonary bypass (CPB) with standard aortic and dual-stage (single) venous cannula unless concomitant valve surgery was performed. The driveline was implanted before heparin. All patients were given 5 g Aminocaproic acid bolus over 10 minutes and an additional 1 g/h for the duration of the operation. Baseline-activated clotting time (ACT) was obtained on all patients who were given 400 U/kg heparin and ACT was maintained greater than 450 seconds throughout the course of the bypass run. At the end of the implantation, all patients were corrected with protamine by titration to return to baseline ACT. The LVAD was enveloped in a GoreTex membrane, and the sternum was closed. Sternum was closed by sternal wire cerclage. A minimum of 3 chest tubes/drainage catheters were placed in all patients.

### Outcomes

The primary outcome of the study was IOB. The secondary outcome is right ventricle (RV) failure and 90-day mortality.

### Statistical analysis

Continuous variables are presented as means and analyzed by Student’s *t*-test. Categorical variables are presented as frequency and percentage and analyzed with chi-square test. Overall survival was evaluated using a Cox model for death; variables included in the multivariable model were chosen a priori and included patient characteristics presented in Table 1. Kaplan-Meier curves were drawn for mortality. All significance tests were 2-tailed and conducted at the 5% significance level. Statistical analysis was performed using STATA (College Station, TX).

**Table 1:** Pre-LVAD-Baseline Characteristics and Intraoperative Bleeding

Baseline characteristics	Total	Non–intraoperative bleeding	%/std dev	Intraoperative bleeding	%/std dev	*p*-value
Patients	208	165	79.33%	43	20.67%	
*Demographics*						
Age mean (±SD)	58.39 (12.18)	57.24	12.51	62.81	9.7	<0.01
Male (%)	157 (75.4)	128	77.58%	29	67.44%	0.16
HTN (%)	86 (41.3)	64	38.79%	22	51.16%	0.14
T2DM (%)	83 (39.9)	66	40.00%	17	39.53%	0.95
COPD (%)	23 (11.0)	17	10.30%	6	13.95%	0.49
CKD (%)	81 (38.9)	61	36.97%	20	46.51%	0.25
CVA (%)	17 (8.17)	16	9.70%	1	2.33%	0.11
CAD (%)	88 (42.3)	64	38.79%	24	55.81%	0.04
CABG (%)	30 (14.2)	18	10.91%	12	27.91%	<0.01
PCI (%)	57 (27.4)	38	23.03%	19	44.19%	<0.01
ICM (%)	61 (29.3)	45	27.27%	16	37.21%	0.2
NICM (%)	147 (70.6)	120	72.73%	27	62.79%	
Afib (%)	66 (31.7)	52	31.52%	14	32.56%	0.89
VF/VT (%)	72 (34.6)	56	33.94%	16	37.21%	0.68
BMI (±SD)	29.69	30.06	6.15	28.69	5.52	0.04
ASA (%)	141 (67.7)	106	64.24%	35	81.40%	0.03

Abbreviations: Afib, atrial fibrillation; ASA, aspirin; CABG, coronary artery bypass grafting; BMI, Body Mass Index; CAD, coronary artery disease; CKD, chronic kidney disease; COPD, chronic obstructive pulmonary disease; CVA, cerebral vascular accident; HTN, hypertension; ICM, ischemic cardiomyopathy; LVAD, left ventricular assist device; NICM, nonischemic cardiomyopathy; PCI, percutaneous coronary intervention; T2DM, type 2 diabetes mellitus; VT/VF, ventricular fibrillation/ventricular tachycardia.

## Results

Among a total of 326 patients, 118 patients were excluded. Among 208 patients included in the analysis, 43 (20.67%) had IOB.

### Baseline characteristics

The mean age was 58.39 years, Body Mass Index (BMI) mean was 29.69 kg/m^2^, 75.4% were male, 41.3% had hypertension, 39.9% had T2DM, 11% had COPD, 42.3% had coronary artery disease (CAD), 14.2% had previous coronary artery bypass grafting (CABG), 27.4% had previous PCI, and 70.6% had nonischemic cardiomyopathy. A total of 67.7% of patients were taking aspirin within 5 days before LVAD surgery. The aspirin within 5 days preoperatively was significantly higher among the IOB cohort (64.24% vs 81.40%, *p* = 0.03). (Table 1).

### LVAD indication and INTERMACS profile

Among 208 patients, 51.9% had LVAD HM II, 41.34% HM3, and 6.7% HVAD. The LVAD indication as bridge to transplant was 50.4% and destination therapy was 49.5%. In total patients, 7.2% were Intermacs 1, 70.7% patients were Intermacs profile 2 and 3. Rates of IOB were higher among the groups of patients with an Intermacs profile 1 (23.26% vs 3.03%, *p* < 0.01) and Intermacs profile 2 (32.56% vs 29.7%, *p* < 0.01) (Table 2).

**Table 2:** Left Ventricle Assist Device Brand, Indication, INTERMACS Profile, and Intraoperative Bleeding

Baseline characteristics	Total	Non–intraoperative bleeding	%/std dev	Intraoperative bleeding	%/std dev	*p*-value
*LVAD device*						0.61
HVAD (%)	14 (6.7)	12	7.27%	2	4.65%	
HM II (%)	108 (51.9)	83	50.30%	25	58.14%	
HM3 (%)	86 (41.34)	70	42.42%	16	37.21%	
*LVAD indication*						0.02
Destination therapy (%)	103 (49.5)	75	45.45%	28	65.11%	
Bridge to transplant (%)	105 (50.4)	90	54.55%	15	34.88%	
*INTERMACS*						<0.01
INTERMACS 1 (%)	15 (7.2)	5	3.03%	10	23.26%	
INTERMACS 2 (%)	63 (30.2)	49	29.70%	14	32.56%	
INTERMACS 3 (%)	84 (40.3)	71	43.03%	13	30.23%	
INTERMACS 4 (%)	28 (13.4)	24	14.55%	4	9.30%	
INTERMACS 5 (%)	18 (8.6)	16	9.70%	2	4.65%	

Abbreviations: HM II, HeartMate II; HM3, HeartMate 3; HVAD, HeartWare Ventricular Assist Device; LVAD, left ventricular assist device.

### Intraoperative events

The IOB group had lower hemoglobin (11.67 vs 10.35 mg/dl, *p* < 0.01), higher WBC (8.23 vs 9.27 mg/dl *p* = 0.02), lower platelets (198.2 vs 171.39 mcl *p* = 0.01), higher aspartate aminotransferase (45.03 vs 308.9 U/liter, *p* < 0.01) and low albumin (3.68 vs 3.45 g/dl, *p* = 0.01). The mean left ventricle end-diastolic diameter was 6.64 cm (Table 3).

**Table 3:** Pre-LVAD Laboratory, Left Ventricle Dimensions, Hemodynamics, Temporary Mechanical Circulatory Support, and Intraoperative Bleeding

Baseline characteristics	Total	Non–intraoperative bleeding	%/std dev	Intraoperative bleeding	%/std dev	*p*-value
*Pre-LVAD laboratory*						
Na (±SD)	134.3	133.65	14.56	136.81	5.13	0.08
Creatine (±SD)	1.37	1.35	0.51	1.42	0.42	0.21
Hb (±SD)	11.40	11.67	2.02	10.35	2.1	<0.01
WBC (±SD)	8.44	8.23	2.84	9.27	3.99	0.02
Platelets (±SD)	192.7	198.25	67.53	171.39	68.71	0.01
Bilirubin (±SD)	1.80	1.97	13.01	1.16	0.75	0.34
AST (±SD)	99.58	45.03	86.74	308.9	1372	<0.01
Albumin (±SD)	91.96	3.68	0.47	3.45	0.78	0.01
*Left ventricle dimension*						
LV end-diastolic diameter (cm,±SD)	6.64	6.72	1.01	6.33	0.91	0.01
*Pre-LVAD hemodynamics*						
RA Mean (±SD)	14.18	14.04	6.17	14.72	5.37	0.18
PAS (±SD)	54.36	53.85	1.18	56.30	17.23	0.10
PAD (±SD)	28.09	28.01	8.70	28.40	7.72	0.39
PAM (±SD)	37.95	37.72	10.58	38.80	10.76	0.27
PCWP (±SD)	26.72	26.60	8.71	27.16	6.97	0.35
PVR (±SD)	2.70	2.64	2.39	2.90	2.10	0.26
PAPI (±SD)	2.23	2.22	1.36	2.25	1.62	0.45
CO (±SD)	4.24	4.20	1.19	4.39	1.79	0.21
CI (±SD)	2.03	2.0	0.52	2.11	0.76	0.16
*tMCS pre-LVAD*		165		43		<0.01
Non-tMCS pre-LVAD (%)	123 (59.1)	107	64.84%	16	37.20%	
Any tMCS pre-LVAD (%)	85 (40.8)	58	35.15%	27	62.79%	
IABP (%)	63 (30.2)	49	29.70%	14	32.56%	
Impella CP (%)	3 (1.4)	3	1.82%	3	6.98%	
Impella 5.0/5.5 (%)	10 (4.8)	6	3.64%	4	9.30%	
TANDEM heart (%)	2 (0.9)	1	0.61%	1	2.33%	
VA-ECMO (%)	5 (2.4)	1	0.61%	4	9.30%	
ECPella (%)	1 (0.48)	1	0.61%	1	2.33%	

Abbreviations: AST, aspartate aminotransferase; CI, cardiac index; CO, cardiac output; Hb, hemoglobin; IABP, intra-aortic balloon pump; LV, left ventricle; LVAD, left ventricular assist device; PAPI, pulmonary arterial pulse index; PCWP, pulmonary capillary wedge pressure; PVR, pulmonary vascular resistance; RA, right atrial; tMCS, temporary mechanical assist device; WBC, white blood cells.

### Hemodynamics pre-LVAD

The hemodynamics within 24 hours pre-LVAD implant showed no significant differences among patients with or without IOB. The mean right atrial pressure was 14.18 mm Hg, pulmonary artery (PA) systolic 54.36 mm Hg, PA diastolic 28.09 mm Hg, PA mean 37.95 mm Hg, pulmonary capillary wedge pressure 26.72 mm Hg, PVR 2.7 Woods, pulmonary arterial pulse index 2.23, cardiac output by thermodilution 4.24 liter/min, and cardiac index 2.03 liter/min/m^2^ (Table 3).

### Temporary mechanical circulatory support pre-LVAD

Temporal mechanical circulatory support (tMCS) pre-LVAD had higher frequency of IOB post-LVAD (35.15% vs 62.79%, *p* < 0.01). Impella CP (1.82% vs 6.98%), Impella 5.0/5.5 (3.64% vs 9.3%), TANDEM Heart (0.61% vs 2.33%), VA-ECMO (0.61% vs 9.3%), ECPella (00.61% vs 2.33%). Intra-aortic balloon pump (IABP) had no significant difference IOB post-LVAD (29.70% vs 32.56%) (Table 3).

### Clinical outcomes

IOB occurred in 43 patients (20.67%) while 165 (79.33%) patients did not. Patients who experienced IOB were older (57.24 years SD ± 9.7 vs 62.81 years SD ± 12.51, *p* < 0.01). Also, IOB group had higher prevalence of CAD (38.79% vs 55.81%, *p* = 0.04), CABG (10.91% vs 27.91%, *p* < 0.01), percutaneous coronary intervention (23.03% vs 44.19%, *p* < 0.01) (Table 1).

### Intraoperative events

The IOB cohort required significantly higher units of blood products intraoperatively (packed red blood cells 0.60 vs 7.34 U, platelets 0.64 vs 2.65 U, fresh frozen plasma 1.42 vs 3.44 U, cryoprecipitate 0.64 vs 1.79 U; *p* < 0.01). Also, the bypass time was prolonged 156.56 vs 111.28 minutes, *p* < 0.01. The chest was left open more frequently in the IOB group (19.39% vs 30.23%, *p* = 0.12).

### Perioperative events

Within 24 hours postsurgery, the mean LVAD flow was 5.58 liter/min with no significant difference between groups. Also, the dosage of inotropes and vasopressors was not significantly different among groups. Chest tube output within 24 hours postoperative was higher in the IOB group (856 vs 1518 ml, *p* < 0.01). Chest exploration within 7 days postsurgery was more frequent in the IOB group (9.09% vs 27.91%, *p* < 0.01).

### Perioperative clinical outcomes

IOB was associated with higher rates of right ventricular failure (31.52% vs 53.49%, *p* < 0.01), use of right ventricular mechanical circulatory support (MCS) (right ventricular assist device) (13.33% vs 27.91%, *p* < 0.01), and prolonged post-LVAD inotropes (15.09 vs 20.69 days, *p* = 0.02). This cohort of patients with IOB also had higher rates of 90-day mortality (2.42% vs 23.26%, *p* < 0.01) (Table 4).

**Table 4:** Intraoperative Bleeding, Perioperative Events, Clinical Outcomes

Outcomes	Total	Non–intraoperative bleeding	%/std dev	Intraoperative bleeding	%/std dev	*p*-value
*Intraoperative*						
Bypass time mean (±SD)	120.5	111.28	47.51	156.16	78.54	<0.01
Chest open (%)	45 (21.6)	32	19.39%	13	30.23%	0.12
OR RBCs mean (±SD)	2.0	0.60	0.96	7.34	3.48	<0.01
OR platelets mean (±SD)	1.05	0.64	0.91	2.65	1.42	<0.01
OR FFP mean (±SD)	2.33	1.42	2.22	3.44	4.77	<0.01
OR cryo mean (±SD)	0.88	0.64	0.81	1.79	1.24	<0.01
*Post-LVAD outcomes*						
LVAD flow mean (±SD)	5.58	5.97	12.5	4.02	0.64	0.15
Dobutamine mean (±SD)	1.92	1.94	2.69	1.86	2.73	0.42
Milrinone mean (±SD)	0.17	0.18	0.17	0.15	0.19	0.22
Epinephrine mean (±SD)	0.05	0.05	0.04	0.06	0.06	0.16
Vasopressin mean (±SD)	0.01	0.01	0.02	0.02	0.03	0.01
Norepinephrine mean (±SD)	0.02	0.02	0.03	0.02	0.03	0.40
Chest tube output mean (±SD)	993.4	856.6	597	1518	1522	<0.01
Chest explore within 7 days	27 (12.9)	15	9.09%	12	27.91%	<0.01
RVAD (%)	34 (16.4)	22	13.33%	12	27.91%	0.01
RV failure (%)	75 (36)	52	31.52%	23	53.49%	<0.01
Post-LVAD inotrope mean days (±SD)	16.23	15.09	15.77	20.69	16.70	0.02
*90-day mortality (%)*	14 (6.7)	4	2.42%	10	23.26%	<0.01

Abbreviations: FFP, fresh frozen plasma; LVAD, left ventricular assist device; OR, operating room; RBC, red blood cells; RV, right ventricle; RVAD, right ventricular assist device.

### Predictors of intraoperative bleeding

**Figure 2:**
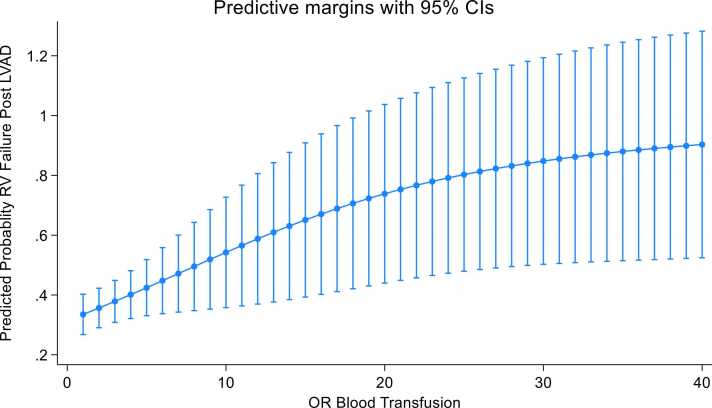
Predicted probability of RV failure post-LVAD and intraoperative blood transfusion. LVAD, left ventricular assist device; OR, operating room; RV, right ventricle.

**Figure 3:**
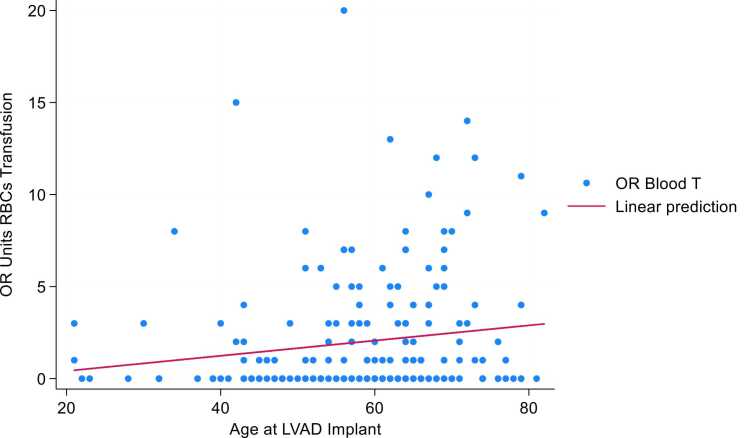
Linear regression model age at LVAD implant and intraoperative red blood cells transfusion. (Coefficient 0.41, *p* < 0.02, CI 95% 0.004-0.07). LVAD, left ventricular assist device; OR, operating room; RBC, red blood cells.

Liner regression model showed a positive correlation between higher red blood cells transfusion during LVAD surgery and predicted probability of RV failure post-LVAD (Figure 2). Liner regression model showed a positive correlation between age and IOB during LVAD implant (coefficient 0.41, *p* < 0.02, CI 95% 0.004-0.07) (Figure 3).

**Figure 4:**
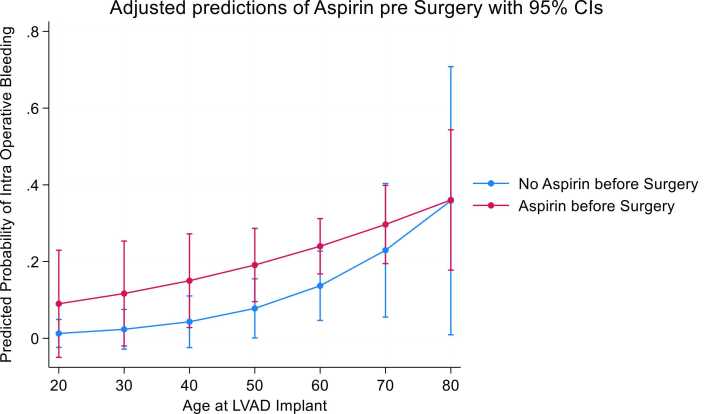
Predicted probability of intraoperative bleeding, aspirin 5 days before surgery, and age at LVAD implant. LVAD, left ventricular assist device.

**Figure 5:**
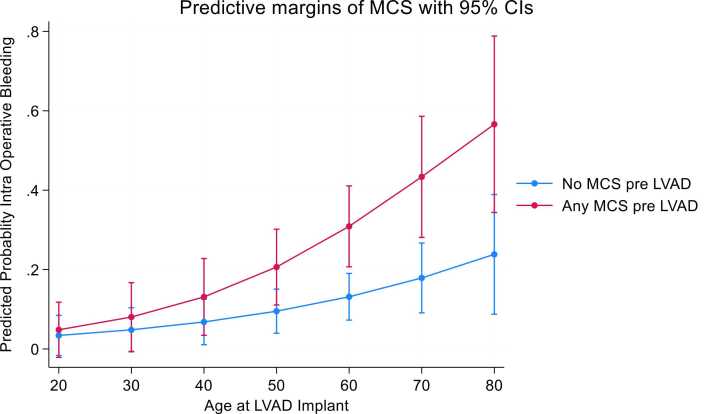
Predicted probability of intraoperative bleeding, mechanical circulatory support pre-LVAD, and age at LVAD implant. LVAD, left ventricular assist device; MCS, mechanical circulatory support.

Adjusted prediction analysis with age at implant showed no significant differences among patients taking aspirin or not before surgery and IOB (Figure 4). Adjusted prediction analysis with age at implant showed significant IOB among patients who were supported by any mechanical support before LVAD who were older than 60 years old (Figure 5).

Univariate analysis showed that high age at implant increases the risk for IOB (OR 1.04, 95% 1.01-1.07, *p* < 0.03), history of CABG increases 3 times the risk of bleeding (OR 3.16, CI 1.38-7.22, *p* < 0.006), as well as any MCS pre-LVAD (OR 3.11, 95% CI 1.55-6.24, *p* < 0.001). Antiplatelets 5 days before surgery increase the 2 times risk for IOB (OR 2.43, 95% CI 1.06-5.59, *p* = 0.03). There was not a statistically significant association between the risk of IOB and the different device types HM3 (OR 0.8, 95% CI 0.40-1.60, *p* = 0.53), HM II (OR 1.37, 95% CI 0.69-2.70, *p* = 0.36), HVAD (OR 0.62, 95% CI 0.13-2.88, *p* = 0.54) (Table 5).

**Table 5:** Univariate and Multivariate Logistic Regression Analysis Intraoperative Bleeding

Logistic regression analysis	Univariate logistic model			Multivariate logistic model		
Characteristics	OR	95% CI	*p-*value	OR	95% CI	*p*-value
Age at implant	1.03	1.01-1.07	0.03	1.03	0.99-1.07	0.09
CABG pre-LVAD	3.16	1. 38-7.22	0.006	2.95	1.18-7.34	0.02
Bridge to transplant	0.44	0.22-0.89	0.02	0.55	0.24-1.26	0.16
Any tMCS pre-LVAD	3.11	1.55-6.24	0.001	3.67	1.71-7.89	0.001
Antiplatelets pre-LVAD	2.43	1.06-5.59	0.03	1.77	0.69-4.51	0.23
HM3	0.80	0.40-1.60	0.53	1.41	0.23-8.63	0.70
HM II	1.37	0.69-2.70	0.36	1.54	0.26-9.09	0.63
HVAD	0.62	0.13-2.88	0.54	Omitted		

Abbreviations: CABG, coronary artery bypass graft; HM II, HeartMate II; HM3, HeartMate 3; HVAD, HeartWare Ventricular Assist Device; LVAD, left ventricular assist device; tMCS, temporary mechanical assist device.

Multilogistic regression analysis showed that age at implant, CABG pre-LVAD, and any tMCS pre-LVAD were independent predictors of IOB during hospitalization (Table 5).

### Predictors of clinical outcomes

**Figure 6:**
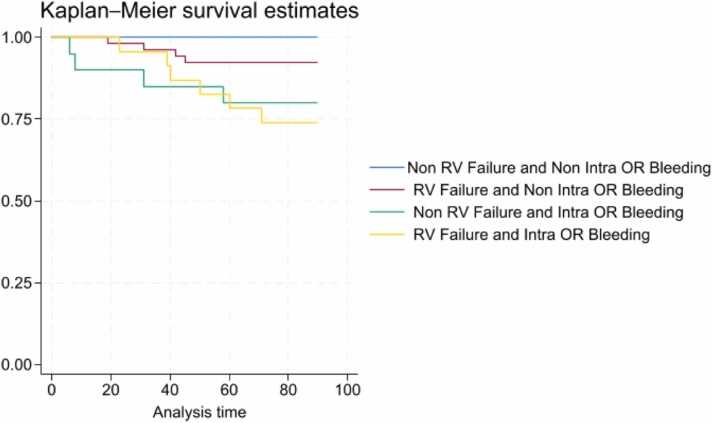
Cox regression with Breslow method for ties. The 90-day survival among patients with RV failure post-LVAD and intraoperative bleeding. RV failure post-LVAD (HR 2.86, *p* = 0.08, CI 95% 0.87-9.40). Intraoperative bleeding (HR 8.24, *p* < 0.01, CI 95% 2.51-27.03). OR, operating room; LVAD, left ventricular assist device; RV, right ventricle.

**Figure 7:**
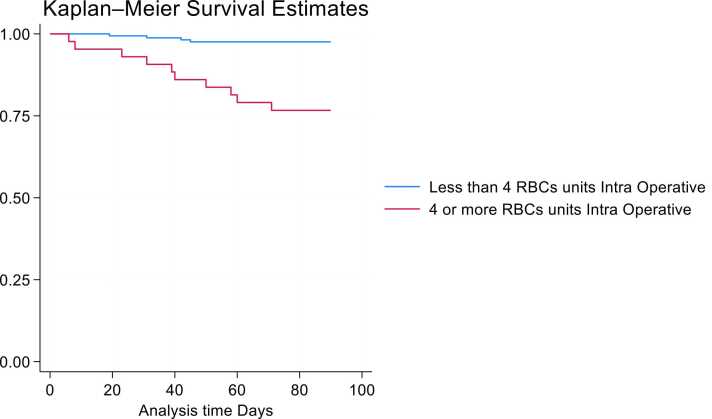
Cox regression model with Breslow ties intraoperative bleeding and 90-day mortality. The 90-day mortality intraoperative bleeding (HR 10.4, *p* < 0.01, CI 95% 3.28-33.38). Two hundred and six subjects with 14 failures. RBC, red blood cells.

Patients with IOB had higher frequency of RV failure post-LVAD (23.2% vs 2.4%, *p* > 0.01) and 90-day mortality (53.4% vs 31.2%, *p* < 0.01). Cox regression model showed that IOB increases significantly 90-day mortality post-LVAD (HR 8.24, *p* < 0.01, CI 95% 2.51-27.03) with or without RV failure post-LVAD (HR 2.86, *p* = 0.08, CI 95% 0.87-9.40) (Figure 6). Kaplan-Meier survival curve estimates decreased significantly in the IOB cohort (Cox regression HR 10.47 [95% CI 3.28-33.39, *p* < 0.01)] (Figure 7).

## Discussion

Our study highlights several key findings regarding IOB in patients undergoing CF-LVAD implantation. The prevalence of IOB in our study was 20.67%. Notably, we identified that advanced age, prior CABG, and preoperative tMCS significantly increase the risk of IOB. These findings are crucial as they underscore the need for careful preoperative assessment and strategic planning to mitigate bleeding risks.

### Platelets count, antiplatelets, and platelets function

Bleeding is the most common adverse event after LVAD implantation observed in 40% to 60% of patients.^1^, ^13^ Bleeding events occurring in the first 2 weeks postoperatively are generally related to the surgery. Common challenges in cardiac surgery include bleeding and transfusion, which are affected by patient comorbidities, the complexity of surgery, hypothermia, and the hemostatic changes of CPB-inducing thrombin generation, fibrinolysis, and platelet dysfunction.^14^, ^15^, ^16^, ^17^ Bleeding, which is prevalent after CF-LVAD implantation in patients with low postoperative platelet counts, is associated with poor prognosis and, as a result, risk stratification using the postoperative platelet count may be beneficial for these patients.^18^ Moreover, survival rate of patients who experienced bleeding was significantly lower at 4 years compared to those without bleeding (73.6% vs 90.1%, *p* = 0.039). A challenging issue before LVAD implantation is the anticoagulation management. Antiplatelet management is less challenging than anticoagulants before LVAD implantation. Typical antiplatelet management in patients who receive antiplatelet medication due to prior stent implantation, CABG or ischemic cardiovascular disease, would be to continue aspirin until the day of surgery.^19^, ^20^ Our study was not designed to compare groups among patients taking aspirin; however, those who had IOB had higher frequency of aspirin 5 days before surgery. Also, IOB group had lower count of platelets. Aspirin was associated with higher IOB in our patients (OR 2.43 95% CI 1.06-5.59, *p* < 0.03). This raises the question of whether stopping aspirin before surgery could decrease perioperative bleeding. Current International Society for Heart and Lung Transplantation (ISHLT) guidelines do not provide specific recommendations for aspirin discontinuation before LVAD implantation surgery. Current Society of Thoracic Surgeons recommendations for antiplatelet management pre-CABG have an IIa recommendation to stop 3 to 5 days before elective surgery to reduce transfusion-related complications.^21^ The American College of Cardiology and American Heart Association guidelines recommend stopping aspirin 7 to 10 days before CABG.^22^ However, the American College of Chest Physicians recommends continuing aspirin, with a recommendation 1C, before CABG.^23^ The reason for these discrepancies and the lack of clear recommendations is the absence of randomized trials using antiplatelets before LVAD surgery. Nevertheless, we think that further analysis should be considered as previous studies suggest that aspirin before cardiac surgery increases bleeding complications.^24^

Early platelet function testing may help guide the timing of cardiac surgery after antiplatelet discontinuation, and the need for targeted therapeutic hemostatic pathway in the preoperative and perioperative periods. However, most recently ISHLT consensus 2024 agreed that no markers of platelets activity test are routinely utilized in patient management due to poor correlation with adverse events in CF-LVAD.^25^

It is well known that previous LVAD pumps available as HVAD and HM II were more susceptible for pump thrombosis with low doses of aspirin or single therapy with warfarin, respectively. Our study has a mixed population with 51% HM II and 41% HM3. The indication of aspirin post-HM3 not only has shown no benefit in reduction of thrombotic events but also increases the risk of bleeding. Having in consideration that IOB could increase the risk for RV failure post-LVAD and mortality as was evidently seen in this study, new consideration of stopping aspirin presurgery should be evaluated in the future within a randomized trial to assess this potential benefit.

### Age and intraoperative bleeding

Higher age was associated with high IOB. In patients who were supported by tMCS, including Impella, Tandem Heart, and ECMO, we found that elders had higher frequency of IOB. These results depend on the level critical illness and severity of cardiogenic shock of these patients. INTERMACs profiles 1 and 2 had the highest frequency of bleeding that proportionally increased with age.

### Blood products

The use of blood products in cardiac surgery is a continuous debate. Transfusion of RBCs has been associated with increased morbidity and mortality.^26^ We found that IOB defined as transfusion with 4 or more RBCs was associated with perioperative right ventricular failure and 90-day mortality. Increased blood product utilization also was associated with prolonged bypass time, high chest tube output within 24 hours postsurgery, higher frequency of chest exploration within 7 days postsurgery. These results emphasize the importance of optimizing perioperative management and developing targeted interventions to improve patient outcomes in this vulnerable population.

### Delayed sternal closure

Clinical practice identifies patients who experience severe, refractory, IOB, and is defined as leaving the operating room with an open or packed chest.^27^ It is occasionally adopted after implantation of LVAD but, through studies, it has not been proven to be beneficial in reducing complications associated with coagulopathy and hemodynamic instability, including cardiac tamponade or right ventricular failure.^28^ Thus, it would be more appropriate to be selectively applied for patients undergoing LVAD implants.^28^

### Cardiopulmonary bypass and bleeding

Several hemostatic changes are induced by CPB. The process ultimately leads to the initiation of the coagulation cascade and lower levels of circulating coagulation factors.^29^, ^30^, ^31^ Additionally, laminar flow promotes the release of proinflammatory cytokines, and longer CPB runs decrease postoperative platelets count and function.^32^ Complement enhances activation of the fibrinolytic pathways^33^ and is associated with increased postoperative bleeding.^34^, ^35^ We did find a weak risk of prolonged CPB and IOB with a modest prolonged bypass time during LVAD implant in the IOB group (111.28 vs 156.16 minutes, *p* < 0.01).

## Limitations

There are some limitations in our study. First, it is a single-center retrospective cohort study including 208 patients implanted with CF-LVAD. The small sample size and the absence of other centers across the United States are significant limitations. Due to this fact, our results may include underpowered analyses that prevent us from making them generalizable for the specific subpopulation. Moreover, due to the fact that randomization is missing, bias may exist in our study due to confounders. As a result, large randomized controlled trials, including more patients from more centers, are required to extract safe conclusions.

IOB during LVAD implant has been poorly evaluated. We evaluated the risk of bleeding during the surgery. Non-adjusted analysis showed an increased risk of bleeding in elders and was significantly increased with previous MCS support. There was some tendency of higher risk of bleeding in elders who took aspirin before surgery. The risk of bleeding adjusted by age, previous CABG, MCS, and aspirin before surgery showed a strong relationship. Previous CABG and any MCS, except IABP, might increase the risk of IOB. Moreover, this study has a mixed population with different brands of LVADs (HVAD, HM II, and HM3) with different surgical techniques that might affect the risk of IOB. This makes it more difficult to find a strong recommendation based on the data of this study.

## Conclusion

Higher age at implant, history of CABG, mechanical circulatory support before the implantation of LVAD and use of antiplatelets 5 days before surgery are independently associated with increased IOB in HF patients undergoing LVAD implantation while, history of CABG, mechanical circulatory support before the implantation of LVAD are independent predictors of IOB during hospitalization in these patients. Moreover, IOB is also correlated with worse clinical outcomes, and specifically, higher frequency of RV failure post-LVAD and higher 90-day mortality.

## Author contributions

Ibrahim Mortada; Writing—Original Draft, Investigation, Data Curation, Writing—Review and Editing. Christos Kourek: Writing—Review and Editing. Rupesh Kshetri: Data Curation. Arun Singhal: Data Curation. Anthony Panos: Data Curation. Alexandros Briasoulis: Data Curation. Mohammed Mhanna: Data Curation. Shareef Mansour: Data Curation. Kristine Yumul: Data Curation. Paulino Alvarez: Data Curation. Ernesto Ruiz Duque: Supervision, Project administration, Writing—Review and Editing, Data Curation, Formal analysis, Methodology, Conceptualization.

## Disclosure statement

The authors declared no conflict of interest.

Funding: None.
